# Functional recovery in a cohort of ECMO and non-ECMO acute respiratory distress syndrome survivors

**DOI:** 10.1186/s13054-023-04724-y

**Published:** 2023-11-14

**Authors:** Mackenzie Snyder, Binta Y. Njie, Ilana Grabenstein, Sara Viola, Hatoon Abbas, Waqas Bhatti, Ryan Lee, Rosalie Traficante, Siu Yan Amy Yeung, Jonathan H. Chow, Ali Tabatabai, Bradley S. Taylor, Siamak Dahi, Thomas Scalea, Joseph Rabin, Alison Grazioli, Carolyn S. Calfee, Noel Britton, Andrea R. Levine

**Affiliations:** 1 University of Maryland School of Medicine, Baltimore, MD USA; 2 Department of Medicine, Division of Critical Care Medicine, University of Maryland Baltimore Washington Medical Center, Baltimore, MD USA; 3 Department of Medicine, Division of Pulmonary and Critical Care Medicine, University of Maryland School of Medicine, 110 S. Paca St, Baltimore, MD 21231 USA; 4 Department of Pharmacy Services, University of Maryland Medical Center, Baltimore, MD USA; 5 Department of Anesthesiology and Critical Care Medicine, The George Washington University School of Medicine, Washington, DC USA; 6 Department of Medicine, Division of Education, University of Maryland School of Medicine, Baltimore, MD USA; 7 Division of Cardiothoracic Surgery, Department of Surgery, University of Maryland School of Medicine, Baltimore, MD USA; 8 Department of Surgery and Program in Trauma, R Adams Crowley Shock Trauma Center, University of Maryland School of Medicine, Baltimore, MD USA; 9 Department of Medicine, University of Maryland School of Medicine, Program in Trauma, Baltimore, MD USA; 10 Division of Pulmonary and Critical Care, Department of Medicine, University of California, San Francisco, CA USA; 11 Department of Medicine, Division of Pulmonary and Critical Care Medicine, Johns Hopkins University School of Medicine, Baltimore, MD USA

**Keywords:** ARDS, ECMO, COVID-19, Pulmonary function tests, Functional recovery, Long-term outcomes

## Abstract

**Background:**

The mortality benefit of VV-ECMO in ARDS has been extensively studied, but the impact on long-term functional outcomes of survivors is poorly defined. We aimed to assess the association between ECMO and functional outcomes in a contemporaneous cohort of survivors of ARDS.

**Methods:**

Multicenter retrospective cohort study of ARDS survivors who presented to follow-up clinic. The primary outcome was FVC% predicted. Univariate and multivariate regression models were used to evaluate the impact of ECMO on the primary outcome.

**Results:**

This study enrolled 110 survivors of ARDS, 34 of whom were managed using ECMO. The ECMO cohort was younger (35 [28, 50] vs. 51 [44, 61] years old, *p* < 0.01), less likely to have COVID-19 (58% vs. 96%, *p* < 0.01), more severely ill based on the Sequential Organ Failure Assessment (SOFA) score (7 [5, 9] vs. 4 [3, 6], *p* < 0.01), dynamic lung compliance (15 mL/cmH_2_0 [11, 20] vs. 27 mL/cmH_2_0 [23, 35], *p* < 0.01), oxygenation index (26 [22, 33] vs. 9 [6, 11], *p* < 0.01), and their need for rescue modes of ventilation. ECMO patients had significantly longer lengths of hospitalization (46 [27, 62] vs. 16 [12, 31] days, *p* < 0.01) ICU stay (29 [19, 43] vs. 10 [5, 17] days, *p* < 0.01), and duration of mechanical ventilation (24 [14, 42] vs. 10 [7, 17] days, *p* < 0.01). Functional outcomes were similar in ECMO and non-ECMO patients. ECMO did not predict changes in lung function when adjusting for age, SOFA, COVID-19 status, or length of hospitalization.

**Conclusions:**

There were no significant differences in the FVC% predicted, or other markers of pulmonary, neurocognitive, or psychiatric functional recovery outcomes, when comparing a contemporaneous clinic-based cohort of survivors of ARDS managed with ECMO to those without ECMO.

**Supplementary Information:**

The online version contains supplementary material available at 10.1186/s13054-023-04724-y.

## Background

Veno-venous extracorporeal membrane oxygenation (ECMO) is a salvage therapy used in part for ARDS patients with severe hypoxemia. To date, there have been nearly 17,000 patients with COVID-19 ARDS placed on ECMO [1]. While the use of ECMO increased as a result of the COVID-19 pandemic, the mortality benefit is widely debated [2–5]. The use of ECMO as a salvage intervention in the most severe ARDS may rescue patients from fatal hypoxemia and mitigate the potential harms of mechanical ventilation [6]. The 2023 European Society of Intensive Care Medicine (ESICM) guidelines on ARDS make a strong recommendation in favor of ECMO in severe COVID-19 and non-COVID-19 ARDS when performed in an ECMO center [7]. However, ECMO remains a limited, resource intensive, and costly resource [2, 4, 8].

Studies of survivors of ARDS have demonstrated that while spirometry and lung volume have normalized by one year in most patients [9], there are persistent impairments in the diffusion capacity of the lung for carbon monoxide (DLCO), six-minute walk test (6MWT), and psychological outcomes for up to five years [10]. Prior studies have explored the functional and pulmonary recovery of ECMO survivors and found them to be comparable to patients with ARDS who were managed without ECMO [11–14]. These studies predate current practice patterns for ARDS including the routine use of corticosteroids for COVID-19 ARDS, efforts to prioritize reductions in sedation, and the overall avoidance of continuous neuromuscular blockade. Additionally, these studies precede the COVID-19 pandemic which has led to overall longer lengths of hospital and ICU stay as well as more prolonged ECMO durations [15].

Given the scarcity, cost, and potential morbidity of ECMO as a resource, it is important to study its outcomes beyond in-hospital mortality to better inform decisions about ECMO allocation and utilization. We report the long-term pulmonary, physical, and neurocognitive recovery of present-day survivors of severe ARDS who received ECMO in comparison with a contemporaneous cohort of patients with ARDS who were not managed on ECMO.

## Methods

This study was performed from January 2020 through January 2023. This study was determined to be exempt by the University of Maryland and R Adams Cowley Shock Trauma Center Internal Review Board (IRB).

### Patient selection

All patients are survivors of critical illness (including ECMO survivors) at the University of Maryland Medical Center (UMMC), R Adams Cowley Shock Trauma Center, and the University of Maryland Baltimore Washington Medical Center (BWMC) and were offered follow-up at either the UMMC or the BWMC post-ICU clinic. The R Adams Cowley Shock Trauma Center and UMMC are large quaternary care hospitals, and BWMC is a tertiary referral center within the University of Maryland Medical System. Patients included in the study encapsulate all patients who either required ECMO or intensive care unit (ICU) care due to a diagnosis of acute respiratory distress syndrome, survived their inpatient hospitalization, and presented for follow-up at the post-ICU clinic during the study period. In light of the newly proposed Global Definition of ARDS, we included patients managed with high flow nasal cannula (HFNC) and patients with an SaO_2_/FiO_2_ ratio ≤ 315 who otherwise met the Berlin definition of ARDS [16–19].

### University of Maryland ECMO criteria

While there were minor variations in the criteria for ECMO cannulation throughout the COVID-19 pandemic, in general, the University of Maryland utilized the following guidelines to determine who should be considered for cannulation: (1) Hypercapnia (PaCO_2_ > 60 mmHg with pH < 7.25 or inability to ventilate the patient with plateau pressure < 30 cmH_2_O) or (2) severe hypoxemia (PaO_2_/FiO_2_ ratio [P/F ratio] < 50 mmHg with FiO2 > 80% FiO_2_ for > 3 h or P/F ratio < 80 mmHg on 80% FiO_2_ for > 6 h despite optimization of mechanical ventilation). Relative contraindications to ECMO included: (1) Age > 60 years old; (2) BMI > 40 kg/m^2^; (3) > 10 days mechanically ventilated; (4) home oxygen requirement; (5) severe neurological injury/insult; (6) terminal disease with low 1-year survival; (7) severe underlying liver disease; (8) acute hepatic failure; (9) Jehovah’s Witness (unwilling to receive blood); (10) Acquired Immune Deficiency Syndrome (AIDS); (11) WBC < 1000 cells/mL^3^ of blood; (12) poor baseline functional status.

### Inpatient hospital stay

Demographics, admission Sequential Organ Function Assessment (SOFA) score, etiology of ARDS, ventilator parameters, vasopressor requirements, laboratory data, inpatient ARDS therapies (i.e., corticosteroids, prone positioning, mechanical ventilation), duration of therapies, and clinical outcomes were extracted from the electronic medical record. Ventilator parameters were extracted within 24 h of arrival to UMMC, Shock Trauma, or BWMC and prior to ECMO cannulation. The worst parameters, defined by the highest PEEP or the need for Airway Pressure Release Ventilation (APRV), within the first 24 h period are reported.

### Follow-up visit

Patients were seen as a standard clinical follow-up visit in the UMMC or BWMC post-ICU clinic approximately three to six months after hospital discharge. Patients were either provided with the clinic contact information at hospital discharge or scheduled for follow-up by discharge coordinators. All clinical evaluations were performed by a board certified pulmonary and critical care provider. Clinical data pertaining to functional recovery was extracted from the patient’s electronic medical record. Pulmonary function testing was conducted in accordance with the standard operating procedure at the UMMC and BWMC clinic and per the discretion of the ordering physician. This testing included pulmonary function tests with or without bronchodilator responsiveness, lung volumes, DLCO, and 6MWT. Cognitive assessment was conducted using the Montreal Cognitive Assessment (MoCA). Post-traumatic stress disorder (PTSD) was evaluated using the PTSD Checklist for DSM-5 (PCL-5). Anxiety and depression were measured using the Hospital Anxiety and Depression Scale (HADS).

### Data analysis

We compared ECMO and non-ECMO patients using the Chi-square test of independence for categorical variables and the Mann–Whitney U test for discrete variables. The primary outcome of interest was Forced Vital Capacity (FVC)% predicted when comparing ECMO to non-ECMO patients. FVC% predicted was chosen as a spirometric surrogate of restrictive lung physiology. The secondary outcomes of interest included spirometry, lung volumes, DLCO, 6MWT, HADS, PCL-5, and MoCA. We conducted univariate and multivariate regression. We fit a univariate model for each clinical covariate and identified the predictors significant at the level of *p* = 0.25 [20]. At each step, variables were added based on p-values, omission of highly colinear variables, as well as clinical and biological plausibility. The Akaiki Information Criterion (AIC) was used to set a limit on the total number of variables included in the final model. We repeated the above analysis limiting our patients to a cohort of COVID-19 patients. We assessed the impact of initial ventilation parameters and pulmonary physiology on FVC% predicted using univariate regression. Two-sided P-values of less than 0.05 were considered to indicate statistical significance except in the case of multiple comparisons. All statistical analyses were performed using R 4.2.2 (R Foundation for Statistical Computing, Vienna, Austria).

## Results

**Fig. 1:**
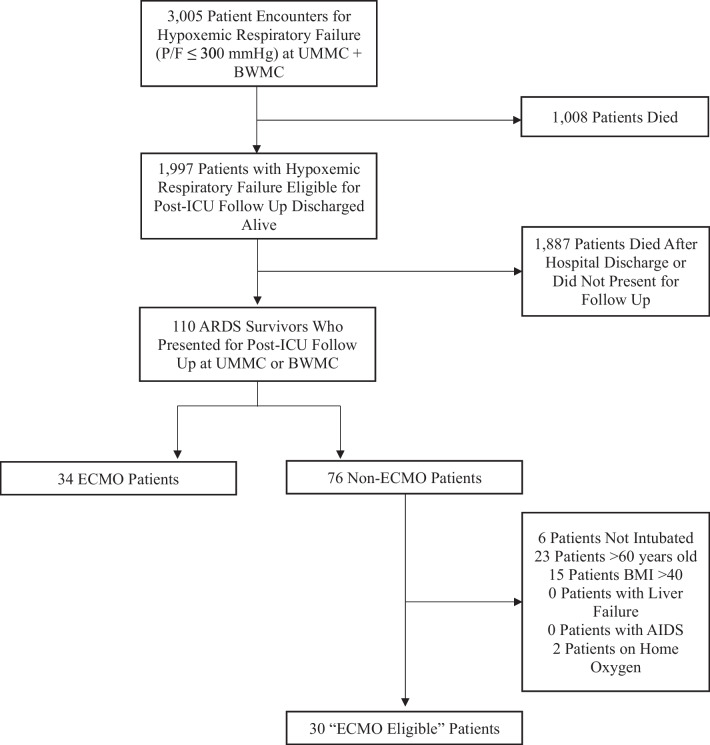
Study consort diagram

From January 2020 through December 2022, there were 3005 patient encounters with a P/F ratio of ≤ 300 mmHg at the University of Maryland Medical Center, R Adams Cowley Shock Trauma Center, and University of Maryland Baltimore Washington Medical Center in the adult Emergency Department or Intensive Care Unit. During the same time period, 211 patients who met the Berlin Criteria for ARDS with refractory hypoxemia were cannulated for VV-ECMO at the University of Maryland Medical Center/R Adams Cowley Shock Trauma Center. Of the 3005 patients with a P/F ratio of ≤ 300 mmHg, 1997 were noted to be alive at the time of discharge, including 141 patients who had been cannulated for ECMO. A total of 110 patients were discharged alive and presented for post-ICU follow-up after requiring VV-ECMO or admission to the ICU for ARDS; these patients represent the focus of this analysis (Fig. 1).

Table 1 describes the population characteristics of these 110 patients on initial presentation to the ICU, the ICU based interventions, and the hospital outcomes. When comparing the ECMO (*n* = 34) to the non-ECMO (*n* = 76) cohort, the ECMO patients were younger with a median age of 35 years old (28, 50) compared to 51 years old (44, 61) in non-ECMO patients (*p* < 0.01). There were no significant differences in sex, race, ethnicity, or BMI between the two groups of survivors. A smaller proportion of patients in the ECMO group had COVID-19 (56%) compared to the non-ECMO group (96%) (*p* < 0.01). The etiology of ARDS in the ECMO cohort is summarized in Additional file 1: Table S1. Patients on ECMO had higher SOFA scores on admission (7 [5, 9] vs. 4 [3, 6], *p* < 0.01) and lower admission PaO_2_/FiO_2_ ratios (104 [81, 158] vs. 150 [103, 210] mmHg, *p* = 0.02). Patients on ECMO were more likely to be on salvage modes of ventilation and had significantly higher peak inspiratory pressures, mean airway pressures, dynamic driving pressures, FiO_2_ requirements, and PaCO_2_. ECMO patients also had a significantly lower dynamic compliance and higher oxygenation index. Significantly more patients in the ECMO group were intubated, received neuromuscular blockade, received inhaled vasodilators, and received vasoactive medications compared to the non-ECMO group. The ECMO group had a longer median duration of hospitalization (46 [27, 62] vs. 16 [12, 31] days, *p* < 0.01), duration of ICU stay (29 [19, 43] vs. 10 [5, 17] days, *p* < 0.01), and days of mechanical ventilation (24 [14, 42] vs. 10 [7, 17] days, *p* < 0.01) (Table 1).

**Table 1:** Baseline population characteristics of patients stratified by ECMO status

	ECMO (*n* = 34)	Non-ECMO (*n* = 76)	*P* value
*Demographics on ICU admission*			
Age, years	35 (28, 50)	51 (44, 61)	< 0.01
Female, *n* (%)	15 (44)	33 (43)	0.95
Race, *n* (%)			0.37
Black or African American	13 (38)	25 (36)	
White	18 (53)	43 (61)	
Asian	3 (9)	2 (3)	
Ethnicity, *n* (%)			0.23
Hispanic or Latino	7 (21)	9 (12)	
Not Hispanic or Latino	27 (79)	67 (88)	
BMI on admission, kg/m^2^	33 (26, 40)	34 (28, 39)	0.78
Smoking history	11 (32)	35 (46)	0.18
Charlson Comorbidity Index, points	0 (0, 1)	2 (1, 3)	< 0.01
Home oxygen requirement, *n* (%)	0 (0)	2 (7)	0.17
COVID-19 positive, *n* (%)	19 (56)	73 (96)	< 0.01
SOFA score*	7 (5, 9)	4 (3, 6)	< 0.01
P/F Ratio on admission, mmHg	104 (81, 158)	150 (103, 210)	0.02
*Initial ventilator parameters on admission to University of Maryland Medical System*			
Ventilator mode, *n* (%)			< 0.01
APRV	11 (38)	0 (0)	
AC/VC	6 (21)	49 (89)	
AC/PC	12 (41)	2 (4)	
PRVC	0 (0)	4 (7)	
Tidal volume, mL	386 (285, 463)	397 (340, 427)	0.40
Peak inspiratory pressure, cmH_2_0	32 (30, 37)	29 (26, 32)	< 0.01
Mean airway pressure, cmH_2_0	24 (21, 27)	18 (15, 20)	< 0.01
Set FiO_2_, %	100 (100, 100)	75 (60, 100)	< 0.01
Dynamic compliance, mL/cmH_2_0	15 (11, 20)	27 (22, 32)	< 0.01
Dynamic driving pressure, cmH_2_0**	26 (19, 30)	15 (12, 18)	< 0.01
Oxygenation Index	26 (22, 33)	9 (6, 11)	< 0.01
pH on ABG	7.30 (7.24, 7.36)	7.36 (7.27, 7.39)	0.06
PaCO_2_, mmHg	55 (47, 70)	47 (42, 52)	< 0.01
Delivered tidal volume by IBW, mL/kg	5.6 (4.9, 6.6)	6.1 (5.8, 6.5)	0.12
Ventilatory ratio	1.8 (1.6, 2.3)	1.7 (1.4, 2.0)	0.11
*Initial ECMO parameters*			
ECMO sweep, L/min	4 (3, 5)	–	–
ECMO flow, L/min	4.9 (4.4, 5.2)	–	–
*ICU Interventions*			
Received corticosteroids, *n* (%)	29 (85)	68 (94)	0.11
Received antibiotics, *n* (%)	31 (91)	64 (86)	0.49
Required mechanical ventilation, *n* (%)	34 (100)	59 (79)	< 0.01
Required proning, *n* (%)	28 (82)	52 (74)	0.36
Required neuromuscular blockade, *n* (%)	29 (85)	46 (64)	0.02
Required inhaled vasodilators, *n* (%)	20 (59)	0 (0)	< 0.01
Required vasoactive drugs, *n* (%)	33 (97)	51 (71)	< 0.01
*Hospital outcomes*			
Length of hospitalization, days	46 (27, 62)	16 (12, 31)	< 0.01
Length of ICU stay, days	29 (19, 43)	10 (5, 17)	< 0.01
Length of mechanical ventilation, days	24 (14, 42)	10 (7, 17)	< 0.01
Discharge location, *n* (%)			0.02
Home	20 (59)	60 (80)	
Rehabilitation (acute or subacute)	14 (41)	15 (20)	

Data are presented as median (Q1, Q3) unless otherwise indicated

*SOFA score excludes GCS, max score is 20

**Dynamic driving pressure is the peak inspiratory pressure minus the PEEP

### Pulmonary function in ECMO- versus non-ECMO-treated patients

**Fig. 2:**
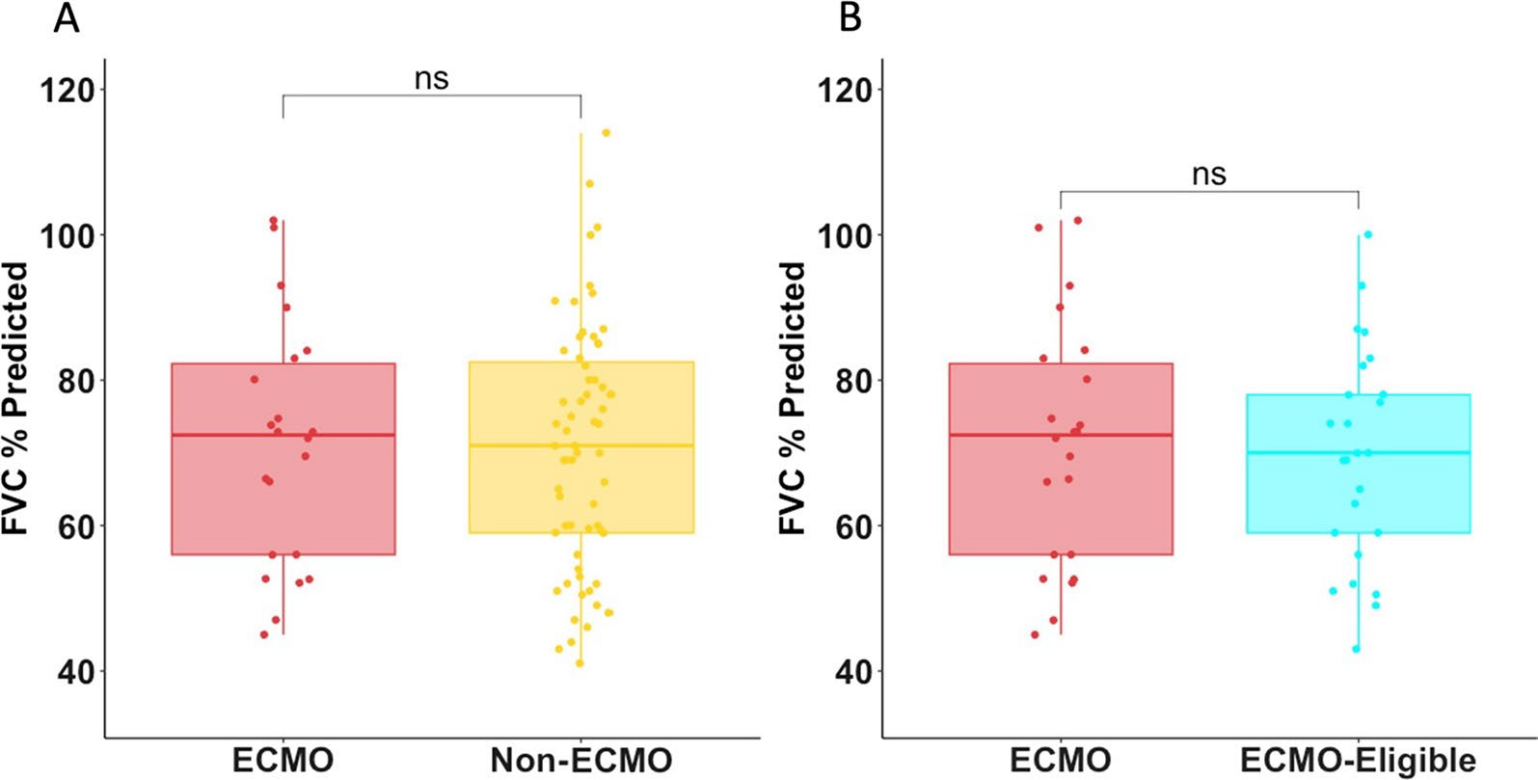
FVC% predicted comparing **A** ECMO (red) to non-ECMO (yellow) patients; **B** ECMO (red) to "ECMO eligible" (teal) patients. ns = non-significant by the Mann–Whitney U test

Pulmonary function tests were obtained, on average, 100 days post-hospital discharge. There was no significant difference in the FVC% predicted between the ECMO and non-ECMO group. Patients in both the ECMO and non-ECMO group demonstrated mild restriction based on FVC% predicted and total lung capacity (TLC). There was a moderate reduction in the DLCO and 6MWT for both the ECMO and non-ECMO cohort. There was no significant difference in any of the spirometry parameters, lung volumes, DLCO or 6MWT when comparing patients managed using ECMO to patients who did not receive ECMO (Table 2, Fig. 2). The use of ECMO did not predict any significant difference in spirometry, lung volumes, DLCO, or 6MWT when compared to non-ECMO in univariate regression analysis. No significant difference in pulmonary function metrics was observed when adjusting for age. Similarly, in multivariate analysis adjusted for age, SOFA, hospital length of stay (LoS), and COVID-19 status, the use of ECMO did not predict any changes in pulmonary recovery when compared to patients who did not receive ECMO. None of the ventilator or pulmonary physiology parameters predicted a significant change in FVC% predicted using univariate regression analysis, when accounting for multiple comparisons (Table 3).

**Table 2:** Differences in spirometry, lung volumes, and six-minute walk test

	Entire cohort			ECMO eligible cohort		
	ECMO (*n* = 34)	Non-ECMO (*n* = 76)	*p* value	ECMO (*n* = 34)	“ECMO Eligible” (*n* = 30)	*P* value
*Primary outcome*						
FVC, % predicted	72 (56, 82)	70 (55, 82)	0.7	72 (56, 82)	70 (57, 78)	0.60
*Secondary outcomes*						
FEV1, L	2.1 (1.8, 2.9)	2.1 (1.5, 2.6)	0.43	2.1 (1.8, 2.9)	2.3 (2.0, 2.9)	0.44
FEV1, % predicted	69 (58, 85)	67 (55, 80)	0.38	69 (58, 85)	70 (57–77)	0.60
FVC, L	2.7 (2.1, 3.4)	2.7 (2.1, 3.4)	0.76	2.7 (2.1, 3.4)	3.1 (2.5, 3.3)	0.46
FEV1/FVC%	84 (79, 87)	80 (76, 86)	0.18	84 (79, 87)	80 (76, 89)	0.67
TLC, L	4.4 (3.3, 5.4)	4.3 (3.4, 5.0)	0.73	4.4 (3.3, 5.4)	4.5 (3.6, 5.9)	0.47
TLC, % predicted	70 (58, 79)	70 (62, 85)	0.75	70 (58, 79)	61 (56, 83)	0.94
DLCO, % predicted	63 (48, 86)	64 (43, 79)	0.49	63 (48, 86)	62 (50, 75)	0.87
6MWT, m	367 (320, 444)	306 (234, 358)	0.12	367 (320, 444)	368 (325, 390)	0.84

Data are presented as median (Q1,Q3) unless otherwise indicated

**Table 3:** Impact of initial ventilator setting and pulmonary physiology on FVC% predicted

Predictor variable	Full cohort			
	Estimate	Confidence Interval	Standard Error	*P* value*
Ventilator mode**				
APRV	10.76	− 1.65 to 23.17	6.21	0.09
AC/PC	4.10	− 9.73 to 17.94	6.93	0.56
PRVC	13.56	− 11.16 to 38.28	12.38	0.28
Ventilatory ratio	3.41	− 5.76 to 12.60	4.6	0.46
Oxygenation index	0.36	− 0.02 to 0.71	0.17	0.04
Inspiratory pressure	− 0.18	− 0.91 to 0.54	0.36	0.61
Dynamic driving pressure	0.10	− 0.49 to 0.70	0.30	0.73
Mean airway pressure	1.03	− 0.18 to 1.89	0.43	0.02
PaCO_2_	0.09	− 0.29 to 0.46	0.19	0.65
Delivered tidal volume	1.40	− 2.48 to 5.29	1.94	0.47

*p value of < 0.00625 is considered significant based on Bonferroni correction

**AC/VC as comparator

### Neurocognitive and psychological recovery in ECMO- versus non-ECMO-treated patients

An exploratory analysis of the neurocognitive and psychological sequelae are reported in Additional file 1: Table S2. A MoCA score of ≥ 26 is normal. The HADS screens for anxiety and depression; a score of ≥ 11 is clinically significant. The PCL-5 is a screening tool used PTSD; the DSM-V uses a cutoff of ≥ 31 to determine probable PTSD. There were no differences in the MoCA, HADS, or PCL-5 scores when comparing the ECMO to the non-ECMO cohort.

### COVID-19 population

The study population included 92 patients who required ECMO or ICU admission due to a diagnosis of COVID-19 ARDS, 19 of whom were managed on ECMO and 73 of whom were not. The patients who received ECMO were significantly younger (43 [30, 52] vs. 52 [44, 62] years old, *p* < 0.01), had a lower Charlson Comorbidity Index (1 point [0, 1] vs. 2 points [1, 3], *p* = 0.04) and a higher SOFA score (6 [5, 8] vs. 4 [3, 6], *p* = 0.01) compared to their non-ECMO counterparts. The COVID-19 ECMO patients had significantly higher ventilator requirements, longer lengths of hospitalization (56 [38, 66] vs. 16 [11, 31] days, *p* < 0.01), ICU stay (40 [27, 61] vs. 10 [5, 17] days, *p* < 0.01), and days requiring mechanical ventilation (35 [27, 46] vs. 10 [7, 16] days, *p* < 0.01) compared to the COVID-19 non-ECMO cohort (Additional file 1: Table S3).

### Pulmonary function in ECMO- versus non-ECMO-treated COVID-19 patients

Among the COVID-19 cohort, ECMO patients had a longer 6MWT distance (381 vs. 294 m, *p* = 0.02). There were no other significant differences in spirometry or lung volumes when comparing the COVID-19 ECMO to the COVID-19 non-ECMO cohort (Additional file 1: Table S4). ECMO did not predict any differences in lung function in a univariate analysis or in analysis adjusting for age. A multivariate analysis, adjusted for age, SOFA, and hospital LoS revealed that ECMO was associated with a 16.4% (CI 1.5–31.4) increase in FEV1% predicted when compared to non-ECMO (*p* = 0.03) which did not meet statistical significance when accounting for multiple comparisons.

### “ECMO eligible” population

In order to compare patients on ECMO to patients of similar age, preexisting medical comorbidities, and severity of acute illness, we identified a sub-group of intubated patients (*n* = 30) who did not meet illness severity to require ECMO but would have potentially been eligible for ECMO cannulation because they did not meet any of the following exclusion criteria: (1) age > 60 years old; (2) BMI > 40 kg/m^2^; (3) history of moderate-to-severe liver failure; (4) history of AIDS; (5) required home oxygen prior to admission (Fig. 1). Compared to these “ECMO eligible” patients, ECMO-treated patients were significantly younger (35 [28, 50] vs. 48 [37, 51] years old, *p* = 0.04), less likely to be COVID-19 positive (56% vs. 90%, *p* < 0.01), and had higher SOFA scores (7 [5, 9] vs. 4 [3, 7], *p* < 0.01). ECMO patients required more ventilator support and had more abnormal pulmonary physiology as evident by their significantly higher inspiratory pressures, mean airway pressures, dynamic driving pressure, ventilator FiO_2_, oxygenation index, PaCO_2_ and a lower PaO_2_ and dynamic compliance (Table 4). All patients were mechanically ventilated, and there was no difference in the use of proning or neuromuscular blockade. ECMO-treated patients more frequently required inhaled vasodilators and vasoactive drugs compared to the “ECMO eligible” group. ECMO patients had a significantly longer length of hospitalization (46 [27, 62] vs. 20 [14, 30] days, *p* < 0.01), ICU stay (29 [19, 43] vs. 12 [10, 15] days, *p* < 0.01), and days requiring mechanical ventilation (24 [14, 42] vs. 10 [8, 13] days, *p* < 0.01) (Table 4).

**Table 4:** Baseline population characteristics of patients who received ECMO compared to those eligible for ECMO

	ECMO (*n* = 34)	“ECMO Eligible” (*n* = 30)	*P* value
*Demographics on ICU admission*			
Age, years	35 (28, 50)	48 (37, 51)	0.04
Female, *n* (%)	15 (44)	10 (34)	0.44
Race, *n* (%)			0.23
Black or African American	13 (38)	8 (32)	
White	18 (53)	17 (68)	
Asian	3 (9)	0 (0)	
Ethnicity, *n* (%)			0.69
Hispanic or Latino	7 (21)	5 (17)	
Not Hispanic or Latino	27 (79)	25 (83)	
BMI on admission kg/m^2^	33 (26, 40)	32 (28–35)	0.33
Smoking history	11 (32)	13 (43)	0.37
Charlson Comorbidity Index, points	0 (0, 1)	1 (0,2)	0.11
COVID-19 positive, *n* (%)	19 (56)	27 (90)	< 0.01
SOFA score*	7 (5, 9)	4 (3, 7)	< 0.01
P/F Ratio on admission, mmHg	104 (81, 158)	163 (85, 233)	0.13
*Initial ventilator parameters on admission to University of Maryland Medical System*			
Ventilator mode, *n* (%)			< 0.01
APRV	11 (38)	0 (0)	
AC/VC	6 (21)	24 (86)	
AC/PC	12 (41)	1 (4)	
PRVC	0 (0)	3 (11)	
Tidal volume, mL	386 (285, 463)	400 (365, 438)	0.39
Peak inspiratory pressure, cmH_2_0	32 (30, 37)	28 (23, 31)	< 0.01
Mean airway pressure, cmH_2_0	24 (21, 27)	17 (14, 19)	< 0.01
Set FiO_2_, %	100 (100, 100)	70 (50, 100)	< 0.01
Dynamic compliance, mL/cmH_2_0	15 (11, 20)	27 (23, 35)	< 0.01
Dynamic driving pressure, cmH_2_0**	26, (19, 30)	14 (12, 17)	< 0.01
Oxygenation index	26 (22, 33)	10 (4, 12)	< 0.01
pH on ABG	7.30 (7.24, 7.36)	7.36 (7.26, 7.41)	0.10
PaCO_2_, mmHg	55 (47, 70)	46 (41, 51)	< 0.01
Delivered tidal volume by IBW, mL/kg	6 (5, 7)	6 (6, 7)	0.13
Ventilatory ratio	1.8 (1.6, 2.3)	1.7 (1.2, 2.0)	0.14
*Initial ECMO parameters*			
ECMO sweep, L/min	4 (3, 5)	–	–
ECMO flow, L/min	5 (4, 5)	–	–
*ICU Interventions*			
Received corticosteroids, *n* (%)	29 (85)	24 (92)	0.40
Received antibiotics, *n* (%)	31 (91)	27 (93)	0.78
Required proning, *n* (%)	28 (82)	23 (82)	0.98
Required neuromuscular blockade, *n* (%)	29 (85)	24 (86)	0.96
Required inhaled vasodilators, *n* (%)	20 (59)	0 (0)	< 0.01
Required vasoactive drugs, *n* (%)	33 (97)	23 (82)	0.05
*Hospital outcomes*			
Length of hospitalization, days	46 (27, 62)	20 (14, 30)	< 0.01
Length of ICU stay, days	29 (19, 43)	12 (10, 15)	< 0.01
Length of mechanical ventilation, days	24 (14, 42)	10 (8, 13)	< 0.01
Discharge location, *n* (%)			0.02
Home	20 (59)	25 (86)	
Rehabilitation (acute or subacute)	14 (41)	4 (14)	

Data are presented as median (Q1,Q3) unless otherwise indicated

*SOFA score excludes GCS, max score is 20

**Dynamic driving pressure defined as peak inspiratory pressure minus PEEP

### Pulmonary function in ECMO versus “ECMO eligible” patients

There was no significant difference in spirometry, lung volumes, DLCO, or 6MWT when comparing the patients who were cannulated for ECMO to the “ECMO eligible” patients (Table 2, Fig. 2). In both univariate and multivariate regression adjusted for age, SOFA, COVID-19 and hospital length of stay, ECMO did not predict any significant differences in spirometry, lung volumes, DLCO, or 6MWT. This lack of association persisted in a sensitivity analysis adjusted for age and COVID-19.

## Discussion

To the best of our knowledge, we report the largest contemporaneous comparison of functional outcomes comparing survivors of ARDS with and without ECMO. We also report a novel direct comparison of patients who were managed using ECMO to patients who were ECMO eligible, based on retrospective assessment, but did not get cannulated for ECMO. Both ECMO and non-ECMO patients had a mild restrictive pattern observed by spirometry at an average of 92 days after hospital discharge. The median FVC% predicted of the non-ECMO cohort was 70% compared to 72% in the ECMO cohort. This finding aligns with prior work by Herridge et al. in survivors of ARDS which demonstrated a FVC% predicted of 72% at three-months [9]. There were no statistically significant differences in any markers of recovery when comparing ECMO to non-ECMO survivors, even when adjusting for age, SOFA, COVID-19 status, and length of hospital stay.

Large, randomized control trials and emulated trials have not demonstrated a mortality benefit from the use of ECMO at 60 or 90 days [2, 15]. However, these analyses are not without limitations, including low sample sizes, high cross-over rates, concern with study designs, and lack of long-term follow-up [4, 6, 9]. There is general agreement that lives have undoubtedly been saved in circumstances when even aggressive modes of conventional ventilation, prone positioning, and neuromuscular blockade could not adequately oxygenate and ventilate patients. However, the longer-term impact of ECMO on more functional outcomes like pulmonary function, anxiety, depression, and PTSD has been less clear. A novel aspect of the presented analysis is the direct comparison of patients on ECMO to those that met eligibility criteria for but were not cannulated for ECMO (“ECMO eligible” cohort). While the sample size of the study precludes the use of propensity score matching methods, the creation of a clearly defined “ECMO eligible” subset in combination with regression modeling adjusting for baseline differences between the two cohorts attempts to minimize the influence of confounding. Interestingly, even with this comparator group, the ECMO-treated patients were significantly sicker based on higher average SOFA scores, the need for salvage modes of mechanical ventilation using higher pressures, higher FiO_2_, impaired CO_2_ clearance, lower lung compliance, longer ICU stay, hospital stay, and an increased utilization of inhaled vasodilators and vasopressors. Each of these factors has the potential to impair recovery. It might be expected, then, that this sicker ECMO population would have worse functional recovery. Our findings, however, suggest that the functional recovery of patients who required ECMO was similar to that of the non-ECMO patients, despite the ECMO patients being more severely ill.

To the best of our knowledge, this is the first study to compare pulmonary, psychiatric, and neurocognitive function of ECMO patients to a contemporaneous cohort of non-ECMO survivors of ARDS since the work published by Grasselli et al. which enrolled patients from 2013 to 2015 [12]. This study is also the first to our knowledge to include both COVID-19 and non-COVID-19 patients. There have been important changes to the management of ARDS in the past decade, including the tendency for lighter sedation, less frequent use of neuromuscular blockade, and the standard use of corticosteroids (in COVID-19 patients). The use of a contemporaneous non-ECMO cohort ensured that both groups were exposed to the same ARDS clinical practice patterns as well as the same COVID-19 related lockdowns, masking policies, vaccine access, and resource limitations. Our study included a diverse population of patients, comprised of nearly 36% Black and 59% White patients, 43% female, and 15% Hispanic or Latino population. This diversity increases the generalizability of our findings to populations of patients known to be more severely afflicted by COVID-19 and to have worse mortality from ARDS and frequently underrepresented in the literature [21]. The University of Maryland is an urban quaternary care center. As a safety net hospital, the University cares for patients who are marginalized–frequently uninsured, lacking primary care, low-income, and highly vulnerable with innumerable barriers to medical care. These social determinants of health may increase the risk for poor outcomes. Yet, the findings of this study demonstrate that most survivors of ARDS (both ECMO and non-ECMO) exhibited only a mild to moderate impairment in pulmonary function and no significant neurocognitive or psychiatric impairment at hospital follow-up despite the fact that these patients often have a paucity of resources and experience barriers to longitudinal care after hospital discharge.

FVC% predicted was used as the primary outcome as a surrogate of restrictive lung physiology. There is no difference in the FVC% predicted between the ECMO group when compared to either the non-ECMO or the “ECMO eligible” group, suggesting that this group does not have more severe restrictive lung disease. However, it is important to consider that the ECMO cohort is consistently significantly younger than the non-ECMO cohort. Thus, if this degree of mild restriction persists, ECMO patients will carry the burden of this morbidity for more of their working, reproductive, and functional lifetime when compared with the older non-ECMO or “ECMO eligible” cohort [21, 22]. Work by Herridge et al. demonstrated normalization of FVC% predicted by six months in survivors of ARDS [20]. Thus, longitudinal follow-up is needed to assess whether this trajectory is seen in ECMO patients.

This study has some limitations, the most important of which is its observational nature. The initial allocation of ECMO to younger patients with fewer comorbidities and contraindications to cannulation who are often earlier in the disease course reveals an inherent selection bias which we can only partially adjust for in our regression analysis. There was additional selection bias in that this study only included individuals who were still alive and who had the ability, resources and desire to complete outpatient long-term follow-up and functional testing, potentially missing both the sickest and most disabled patients and the most recovered patients. Without knowing the demographics of all the patients who died on ECMO or with ARDS, our study may be subject to differential survivorship bias. This study was conducted in an academic medical center which does a high volume of ECMO and cares for a large number of ARDS patients. Improved post-ECMO outcomes have been reported in centers with higher volumes [23], so these data may overestimate the functional recovery of a broader ECMO population. Spirometry is a unidimensional outcome which can be insensitive in detecting restriction, may be impacted by ICU acquired weakness, and which does not quantify global functional impairment or necessarily translate to quality of life. We did not obtain ventilator parameters of our patients after ECMO cannulation. A presumed benefit of ECMO is the use of “ultra low-volume ventilation” which is thought to spare additional ventilator induced lung injury (VILI) in patients with poor lung compliance [24, 25]. Whether this strategy was utilized and how this contributes to pulmonary function at follow-up is not known. The majority of the non-ECMO patients (96%) had COVID-19. No data were available on the variant of SARS CoV-2 that the COVID-19 patients were infected with. Different variants resulted in varying degrees of ARDS severity [26]. The precise impact of COVID-19 on pulmonary recovery after ARDS is unknown, but recent work from Hodgson et al. showed no significant difference in new six-month disability, quality of life, neurocognitive, or psychiatric function when comparing survivors of COVID-19 to non-COVID-19 ARDS [27]. Finally, this study was limited by its relatively small sample size as well as missing data, particularly with regard to some details pertaining to outside hospitalizations and the neurocognitive and psychiatric data. Given the high mortality for ARDS and accounting for tremendous loss to follow-up ICU recovery clinics, the population of ARDS survivors is limited.

## Conclusion

In this sample of 110 contemporary patients with ARDS who were able to come to ICU follow-up clinic, we did not identify any differences in functional recovery when comparing ARDS survivors who were managed on ECMO to patients who did not receive ECMO. ECMO patients were younger but also had significantly more ventilator requirements and pulmonary physiology derangements prior to cannulation, had significantly longer duration of mechanical ventilation, ICU and hospital length of stay, use of neuromuscular blockade, inhaled vasodilators, and vasopressors. These findings are reassuring regarding the impact of this resource intensive therapy. Future studies should focus on enrolling more patients to ensure adequate power and on longitudinal multidimensional assessments of morbidity. Furthermore, future ECMO randomized control trials should look beyond in-hospital mortality as the outcome of interest and consider evaluating patients’ overall recovery, return to work, and quality of life to better determine which patients would receive the greatest overall benefit from ECMO.

### Supplementary Information

**Additional file 1: Table S1** Etiologies of ARDS in ECMO patients. **Table S2** Psychological Health Outcomes. **Table S3** Baseline Population Characteristics of COVID-19 Patients Stratified by ECMO Status. **Table S4** Differences in Spirometry, Lung Volumes, and Six-Minute Walk Test in the COVID-19 Patients.

## Data Availability

The datasets used and/or analyzed during the current study are available from the corresponding author on reasonable request.

## Abbreviations

6MWT: Six-minute walk test
APRV: Airway pressure release ventilation
AC/PC: Assist control/pressure control
AC/VC: Assist control/volume control
ARDS: Acute respiratory distress syndrome
BMI: Body Mass Index
BWMC: Baltimore Washington Medical Center
DLCO: Diffusion capacity of carbon monoxide
ECMO: Extracorporeal membrane oxygenation
FEV1: Forced expiratory volume in 1 s
FVC: Forced vital capacity
HADS: Hospital anxiety and depression scale
IBW: Ideal body weight
ICU: Intensive care unit
LoS: Length of stay
MoCA: Montreal cognitive assessment
OI: Oxygenation Index
P/F ratio: PaO_2_/FiO_2_ ratio
PFT: Pulmonary function test
PTSD: Post-traumatic stress disorder
S/F ratio: SaO_2_/FiO_2_ ratio
SOFA: Sequential organ failure assessment
TLC: Total lung capacity
UMMC: University of Maryland Medical Center
VILI: Ventilator induced lung injury
VR: Ventilatory ratio
VV: Veno-venous
