# Supplementary material for: Functional recovery in a cohort of ECMO and non-ECMO acute respiratory distress syndrome survivors
Source: Crit Care. 2023 Nov 14;27:440. doi: 10.1186/s13054-023-04724-y (PMC10644522; doi:10.1186/s13054-023-04724-y)
Supplement: Supplementary file 1 — Additional file 1: Table S1 Etiologies of ARDS in ECMO patients. Table S2 Psychological Health Outcomes. Table S3 Baseline Population Characteristics of COVID-19 Patients Stratified by ECMO Status. Table S4 Differences in Spirometry, Lung Volumes, and Six-Minute Walk Test in the COVID-19 Patients. [file 13054_2023_4724_MOESM1_ESM.docx]

**Table S1.** Etiologies of ARDS in ECMO patients

| **Etiologies of ARDS** | **n=34 (%)** |
| --- | --- |
| Bacterial Pneumonia | 1 (3) |
| Influenza | 4 (12) |
| COVID-19 | 19 (56) |
| Aspiration | 2 (6) |
| Trauma | 2 (6) |
| Extrapulmonary | 1 (3) |
| Other | 5 (15) |

| \| **Table S2.** Psychological Health Outcomes. \| \| \| \| --- \| --- \| --- \| \|  \| **ECMO (*n*=34)** \| **Non ECMO (*n*=76)** \| **P value** \| \| **MOCA Score^#^** \| 28 (26, 29) \| 26 (24, 27) \| 0.23 \| \| **HADS Score^§^** \| 5 (2, 6) \| 5 (2,11) \| 0.49 \| \| **PCL-5 Score^†^** \| 4 (2, 11) \| 6 (1, 15) \| 0.87 \| \|  \|  \|  \|  \| |  |  |  |
| --- | --- | --- | --- | --- | --- | --- | --- | --- | --- | --- | --- | --- | --- | --- | --- | --- | --- | --- | --- | --- | --- | --- | --- | --- | --- | --- |

Data is presented as median (Q1-Q3) unless otherwise indicated.

^#^MOCA score ≥26 is considered normal, 18-25 indicates mild cognitive impairment, 10-17 moderate cognitive impairment, and < 17 severe cognitive impairment.

**^§^HADS Score is an aggregate of the anxiety and depression score. Maximum anxiety score is 21 and maximum depression score is 21. Scores of 0-7 in subscales (anxiety or depression) are normal, 8-10 borderline, and ≥ 11 are indicative of clinical caseness.**

**^†^PCL-5 score of ≥ is considered abnormal and concerning for Post-Traumatic Stress Disorder (PTSD)**

**Table S3:** Baseline Population Characteristics of COVID-19 Patients Stratified by ECMO Status.

|  | **ECMO (*n*=19)** | **Non-ECMO (*n*=73)** | **P value** |
| --- | --- | --- | --- |
| **Demographics on ICU Admission** | | | |
| Age, years | 43 (30, 52) | 52 (44, 62) | < 0.01 |
| Female, n (%) | 9 (47) | 31 (42) | 0.7 |
| Race, n (%) |  |  | 0.17 |
| Black or African American | 9 (47) | 23 (34) |  |
| White | 8 (42) | 42 (63) |  |
| Asian | 2 (11) | 2 (3) |  |
| Ethnicity, n (%) |  |  | 0.13 |
| Hispanic or Latino | 5 (26) | 9 (12) |  |
| Not Hispanic or Latino | 14 (74) | 65 (88) |  |
| BMI on Admission kg/m^2^ | 35 (28, 42) | 34 (29, 39) | 0.46 |
| Smoking history | 5 (26) | 33 (45) | 0.14 |
| Charlson Comorbidity Index | 1 (0-1) | 2 (1-3) | 0.04 |
| Home Oxygen Requirement, n (%) | 0 (0) | 2 (8) | 0.28 |
| SOFA Score* | 6 (5-8) | 4 (3-6) | 0.01 |
| P/F Ratio on Admission, mmHg | 120 (86, 151) | 150 (104, 210) | 0.06 |
| **Initial Ventilator Parameters on Admission to University of Maryland Medical System** | | | |
| Ventilator Mode, n (%) |  |  |  |
| APRV | 6 (35) | 0 (0) | < 0.01 |
| AC/VC | 6 (35) | 48 (92) |  |
| AC/PC | 5 (29) | 1 (2) |  |
| PRVC | 0 (0) | 3 (6) |  |
| Tidal Volume, mL | 353 (268, 399) | 398 (340, 431) | 0.10 |
| Inspiratory Pressure, cmH_2_0 | 31 (30, 37) | 29 (25, 32) | 0.06 |
| Mean Airway Pressure, cmH_2_0 | 22 (20, 26) | 18 (15, 20) | <0.01 |
| Set FiO2, % | 100 (90, 100) | 80 (60, 100) | <0.01 |
| Dynamic Compliance, mL/cmH_2_0 | 15 (12, 16) | 27 (23, 32) | <0.01 |
| Dynamic Driving Pressure, cmH_2_0** | 24 (19, 30) | 15 (12, 17) | <0.01 |
| Oxygenation Index | 26 (20, 30) | 8 (6, 11) | <0.01 |
| pH on ABG | 7.32 (7.28, 7.37) | 7.36 (7.28, 7.39) | 0.33 |
| PaCO_2_, mmHg | 53 (48, 56) | 46 (42, 52) | <0.01 |
| Delivered Tidal Volume by IBW, mL/kg | 5.37 (4.82, 5.92) | 6.12 (5.78, 6.51) | <0.01 |
| Ventilatory Ratio | 1.84 (1.66, 2.24) | 1.67 (1.36, 2.00) | 0.15 |
| **Initial ECMO Parameters** |  |  |  |
| ECMO Sweep, L/min | 4.5 (3.5, 5.7) | - | - |
| ECMO Flow, L/min | 4.9 (4.4, 5.1) | - | - |
| **ICU Interventions** | | | |
| Received Corticosteroids, n (%) | 18 (95) | 67 (94) | 0.95 |
| Received Antibiotics, n (%) | 17 (90) | 61 (86) | 0.69 |
| Required Mechanical Ventilation, n (%) | 19 (100) | 56 (78) | 0.02 |
| Required Proning, n (%) | 17 (89) | 49 (73) | 0.14 |
| Required Neuromuscular Blockade, n (%) | 18 (95) | 45 (65) | 0.01 |
| Required Inhaled Vasodilators, n (%) | 9 (47) | 0 (0) | < 0.01 |
| Required Vasoactive Drugs, n (%) | 19 (100) | 49 (71) | < 0.01 |
| **Hospital Outcomes** | |  |  |
| Length of Hospitalization, days | 56 (38, 66) | 16 (11, 31) | < 0.01 |
| Length of ICU stay, days | 40 (27, 61) | 10 (5, 17) | < 0.01 |
| Length of Mechanical Ventilation, days | 35 (27, 46) | 10 (7, 16) | < 0.01 |
| Discharge Location, n (%) |  |  | 0.03 |
| Home | 11 (58) | 59 (82) |  |
| Rehabilitation (Acute or Subacute) | 8 (42) | 13 (18) |  |

Data is presented as median (Q1,Q3) unless otherwise indicated.

*SOFA score excludes GCS, max score is 20

**Dynamic Driving Pressure is the Peak Inspiratory Pressure minus the PEEP

**Table S4.** Differences in Spirometry, Lung Volumes, and Six-Minute Walk Test in the COVID-19 Patients.

|  | **ECMO (*n*=19)** | **Non-ECMO (*n*=73)** | **p value** |
| --- | --- | --- | --- |
| **Primary Outcome** | | | |
| **FVC, % predicted** | **70 (54, 79)** | **70 (56, 82)** | **0.87** |
| **Secondary Outcomes** | | | |
| **FEV1, Liters** | **2.1 (1.9, 2.5)** | **2.1 (1.5, 2.6)** | **0.65** |
| **FEV1, % predicted** | **69 (59, 85)** | **68 (55, 80)** | **0.55** |
| **FVC, Liters** | **2.6 (2.1, 3.1)** | **2.7 (2.1, 3.3)** | **0.79** |
| **FEV1/FVC%** | **85 (81, 87)** | **80 (75, 86)** | **0.05*** |
| **TLC, Liters** | **3.4 (3.1, 4.9)** | **4.4 (3.4, 5.1)** | **0.17** |
| **TLC, % predicted** | **64 (54, 79)** | **73 (62, 85)** | **0.35** |
| **DLCO, % predicted** | **63 (50, 83)** | **66 (41, 80)** | **0.42** |
| **6MWT (meters)** | **381 (356, 448)** | **294 (234, 341)** | **0.02*** |

Data is presented as median (Q1-Q3) unless otherwise indicated.

*p value of < 0.00625 is considered significant based on Bonferroni correction for multiple comparisons
